# Comparison of Tonovet® and Tonovet plus® tonometers for measuring intraocular pressure in dogs, cats, horses, cattle, and sheep

**DOI:** 10.14202/vetworld.2024.384-388

**Published:** 2024-02-16

**Authors:** Liga Kovalcuka, Aija Mālniece, Jana Vanaga

**Affiliations:** Clinical Institute, Faculty of Veterinary Medicine, Latvia University of Life Sciences and Technologies, Jelgava, LV-3004, Latvia

**Keywords:** cats, cattle, dogs, horses, sheep, TonoVet plus, TonoVet

## Abstract

**Background and Aim:**

Reference ranges for intraocular pressure (IOP) in healthy animals are device-specific; therefore, it is strongly recommended to use appropriate reference values according to the device. Therefore, our aim was to compare IOP readings made by TonoVet® and TonoVet Plus® in healthy dogs, cats, sheep, cattle, and horses. We compared IOP values measured by TonoVet® and TonoVet Plus® tonometers in clinically normal eyes of dogs, cats, horses, cattle, and sheep.

**Materials and Methods:**

Five groups comprising 20 animals each of dogs (various breeds, 9 months–10 years old, 14 females, 6 males), cats (various breeds, 6 months–12 years old, 8 females, 12 males), horses (various breeds, 5–12 years old, 12 females, 8 males), cattle (Holstein, 1–7 lactation, female), and sheep (Latvian Darkhead ewes, 1–8 years old) were included in the study. Both eyes of all animals were subjected to ophthalmic examination, including evaluation of IOP by rebound tonometry using TonoVet® and TonoVet Plus® devices. Normality was determined using the Shapiro–Wilk test. The independent t-test was used to determine differences between IOP values in the right and left eyes and between both tonometers. This study was approved by the Ethical Commission of the Latvia University of Life Sciences and Technologies (Nr. LLU_Dzaep_2022-2-4).

**Results:**

No differences in IOP between the right and left eyes were found in all cases (p > 0.05). The mean IOP ± standard deviation values in both eyes for TonoVet® and TonoVet Plus® tonometers were as follows: for dogs, 15.25 ± 2.73 mmHg and 19.65 ± 3.46 mmHg; and in cats, 18.88 ± 3.98 mmHg and 18.78 ± 4.26 mmHg, respectively. In horses, mean IOP was 22.15 ± 3.74 mmHg and 24.28 ± 3.00 mmHg; in cattle, 24.73 ± 2.89 mmHg and 23.28 ± 2.97 mmHg; and in sheep, 18.05 ± 3.54 mmHg and 22.49 ± 4.66 mmHg, respectively. Significant differences in IOP values were observed between the tonometers in sheep, dog, and horse groups (mean difference –4.40, –4.48, and 2.13, respectively).

**Conclusion:**

This study showed significantly higher IOP values measured by the TonoVet Plus® tonometer in dogs and sheep.

## Introduction

Tonometry is a basic, standard diagnostic method in veterinary ophthalmology, where accurate and reliable intraocular pressure (IOP) measurement is vital for monitoring glaucoma – a condition characterized by a high IOP and uveitis; corneal perforation with low IOP; and the post-intraocular surgery period [1–3].

IOP results from the balance between aqueous humor production and its outflow in the eye [1, 2] and can be measured using various tonometers. The older generation Schiotz tonometer is an impression (invaginating) tonometer and is no longer commonly used in veterinary practice. In addition, it is not easy to use in active or aggressive small animals and more so in farm animals. In practice, the use of applanation (flattening) Perkins and Tono-Pen XL tonometers can still be found. However, local anesthesia should be used during measurements. In addition, these methods are not ideal for active or non-cooperative animals. More frequently, rebound (impaction) tonometers TonoVet® (TonoVet®, Tiolat Ltd., Vantaa, Finland) or TonoVet Plus® tonometers are used in veterinary practice [4]. These tonometers are advantageous as there is no need to use local anesthesia and the methods are comfortable to use in active and large animals. Furthermore, TonoVet® has a specific calibration setting for dogs and horses, and TonoVet Plus® has specific calibration settings for cats, dogs, rabbits, and horses, thus giving more precise measurement values [1, 2].

Veterinarians are familiar with the normal ranges of IOP values, but attention should be paid to what tool values are discovered because values are not always directly comparable.

IOP ranges can be found in literature for TonoPet® (New York, USA), TonoVet®, and recently some reports for TonoVet Plus®, mainly in dogs and cats [4, 5]. Because previously published reference ranges for IOP in healthy animals are device-specific, using any of the devices, it is strongly recommended to use appropriate reference values according to the device.

Therefore, our aim was to compare IOP readings made by TonoVet® and TonoVet Plus® in healthy dogs, cats, sheep, cattle, and horses. The results of this study will improve data on normal diagnostic values of IOP in cats, dogs, sheep, cattle, and horses according to the TonoVet® or TonoVet Plus® device used.

## Materials and Methods

### Ethical approval and informed consent

The study was approved by the Ethical Commission of the Latvia University of Life Sciences and Technologies (Nr. LLU_Dzaep_2022-2-4), and the animal ethics board approved that General Latvian Republic Food and Veterinary Service animal ethics permission is not necessary. All dogs, cats, sheep, and horses examined were privately owned and informed consent was obtained from the owners. The cattle used in this study were owned by the university teaching farm. The examination procedures performed during routine clinical and ophthalmology examinations did not exceed good veterinary practice principles and were not painful.

### Study period and location

The study was performed from September to December 2022, approximately at the same time period of day (10 am–4 pm) at the Latvia University of Life Sciences and Technologies, Faculty of Veterinary Medicine, University veterinary clinic and university teaching farm “Vecauce.”

### Animals and study design

The study involved 100 animals (200 eyes): 20 healthy dogs (various breeds, 9 months–10 years old, 14 females and 6 males), 20 cats (various breeds, 6 months–12 years old, 8 females, 12 males), 20 sheep (Latvian Darkhead, ewes, 1–8 years old), 20 cattle (Holstein, 1–7 lactation, female), and 20 horses (various breeds, 5–12 years old, 12 females and 8 males) (Table-1).

**Table-1:** Animals used in the study.

Species	Breed	Age	Average age ± SD (years)	Sex		Weight ± SD (kg)

				Female	Male	
Dog	Labrador Retriever (3), Cavalier King Charles Spaniel (2), French bulldog (2), Yorkshire terrier (2), Miniature dachshund, Papillon, Cane Corso, Samoyed, German spitzes, Hovawart, Rottweiler, Beagle, Hungarian vizsla, Bavarian Mountain hound (1)	10 months– 10 years	3.6 ± 3.1	14	6	18.98 ± 10.75
Cat	Non-breed (12), Maine Coon (2), Scottish Fold, Sphynx, Devon Rex, British Shorthair, Burmese, Oriental cat (1)	6 month– 12 year	4.5 ± 3.7	8	12	4.4 ± 1.4
Sheep	Latvian darkhead (20)		3.3 ± 1.71	20	0	71.7 ± 11.4
Horse	Latvian warmblood (7) Lithuanian warmblood (4) Dutch warmblood (3) Latvian draft, Shetland pony (2) Belgian warmblood, Sport pony (1)		11.9 ± 5.15	12	8	541.6 ± 152.4
Cattle	Holstein (20)	1–7 lactation	2.5 ± 1.5	20	0	681.13 ± 61.0

SD=Standard deviation

The selection of the animals was random. Clinically healthy dogs, cats, and horses from pre-breeding examinations and veterinary checks were used in this study. Ewes and cattle were randomly selected by sending all animals through farm electronic scales. Every third animal was selected, and an ophthalmic examination was performed. Animals with epiphora, lacrimation, eye discharge, blepharospasm, or any other signs of clinical ophthalmic or systemic disease were excluded from the study, and the next animal was selected for testing. Each animal was individually examined in a restraining box. During IOP measurements, the animals were gently handled to avoid any tension on the animal neck, which might have influenced the IOP.

All animals in this study underwent complete ophthalmological examination performed by the same person to ensure that they were ophthalmologically healthy. The clinical examination included signaling (animal breed, age, and sex). The ocular examination included basic neurological tests (Menace and Dazzle test, pupillary light reflex). Continuously, direct ophthalmoscopy (Keeler Practitioner, Windsor, UK), monocular ophthalmoscopy using a *PanOptic* ophthalmoscope (Welch Allyn, Romford, UK), and slit-lamp biomicroscopy (Kowa SL15, Nagoya, Aichi, Japan) were performed.

Tonometry was performed by the same person. At the beginning of the study, all IOP measurements were obtained using a calibrated rebound tonometer (TonoVet®, Tiolat Ltd.) using the (d) “dog” calibration setting for dogs, cats, and sheep. Calibration (h) “horse” was used for horses and cattle. A second measurement was performed using a new generation TonoVet Plus® rebound tonometer (TonoVet Plus® Tiolat Ltd.) using the (d) calibration for dogs and sheep, “cat” (c) calibration for cats, and (h) calibration for horses and cattle. Each measurement recorded was the automatically generated average of five successive readings. The use of topical anesthesia is not required when using this tonometer, which benefits the animals since some authors have reported that corneal endothelial and systemic toxicity can occur with the frequent use of topical anesthetics [6, 7]. A single‐use probe was positioned perpendicular to the corneal surface approximately 4 mm from the central cornea. Care was taken to ensure that no compression of the jugular veins or cervical region occurred during measurement.

All measurements were performed during the daytime (10:00 am–4:00 pm) to minimize the effects of changing light conditions on the IOP at different times of day [8, 9].

### Statistical analysis

Statistical analysis of the data was performed using Statistical Package for the Social Sciences, version 12.0.0 (SPSS Inc., Chicago, IL, USA) and Microsoft Office Excel, version 2016 (Microsoft Corp, Redmond, USA). The normality of data sets was tested using Shapiro–Wilk and Wilcoxon signed-rank test. p < 0.05 was considered to be statistically significant.

The arithmetic mean values (X), mean ± standard deviation (SD), standard error, mean difference, and the reference interval of IOP were calculated for each eye separately and both eyes together for all animal species. First, a paired sample t-test was used to compare the IOP obtained from the right and left eyes with each device. No significant difference was found in IOP between the right and left eye (p > 0.05). An independent t-test was used to determine statistically significant differences between IOP measurements for both eyes taken with TonoVet® and TonoVet Plus® devices for each animal species and between IOP values taken by TonoVet® and the IOP values taken by TonoVet Plus® for each species.

## Results

During the study, no signs of ocular irritation or pain were detected in any of the animals examined at any point in time. All measurements were performed in the right and left eye. No differences in IOP between the right and left eyes were found in all cases (p > 0.05); therefore, values from both eyes were used for further calculations. Descriptive values and intervals of IOP in all animals are shown in Table-2.

**Table-2:** Descriptive IOP values measured with Tonovet® and Tonovet Plus® from both eyes.

Species	Instrument	Calibration	n	Mean	Standard deviation	Standard error mean	Mean difference	Suggested reference range
Sheep IOP	Tonovet®	D	40	18.05	3.54	0.56	–4.48*	16.92–19.18
	Tonovet Plus®	D	40	22.49	4.66	0.75		21.05–24.00
Cattle IOP	Tonovet®	H	40	24.73	2.79	0.44	–1.13	23.83–25.62
	Tonovet Plus®	H	40	23.60	2.97	0.47		22.65–24.55
Horse IOP	Tonovet®	H	40	22.15	3.74	0.59	–2.13*	20.95–23.35
	Tonovet Plus®	H	40	24.28	3.00	0.47		23.32–25.23
Dog IOP	Tonovet®	D	40	15.25	2.73	0.43	–4.40*	14.38–16.12
	Tonovet Plus®	D	40	19.65	3.46	0.55		18.54–20.76
Cat IOP	Tonovet®	D	40	18.88	3.98	0.63	–0.10	17.60–20.15
	Tonovet Plus®	C	40	18.78	4.26	0.67		17.41–20.14

* There is significant difference between the mean IOP values taken using the TonoVet® and TonoVet Plus® device, IOP=Intraocular pressure

The mean IOP ± SD values for dogs using a TonoVet® tonometer in both eyes were 15.25 ± 2.73 mmHg and 19.65 ± 3.46 mmHg with the TonoVet Plus®, showing significant differences between devices with a mean difference of 4.40 (p < 0.01). In cats, the IOP measured using TonoVet® was 18.88 ± 3.98 but was 18.78 ± 4.26 mmHg with TonoVet Plus®, showing no significant differences. In sheep, the IOP values using TonoVet® were 18.05 ± 3.54 mmHg and 22.49 ± 4.66 mmHg with TonoVet Plus®, showing a mean difference of 4.48 mmHg, which is significantly higher than TonoVet® (p < 0.01). In cattle, the IOP measured using TonoVet® was 24.73 ± 2.79 but was 23.60 ± 2.97 mmHg with TonoVet Plus®, showing no significant differences. In horses, the IOP measured with TonoVet® was 22.15 ± 3.74 but was 24.28 ± 3.00 mmHg with TonoVet Plus®, showing a mean difference of 2.13 mmHg (Table-2).

## Discussion

The current study was undertaken to compare the normal values of IOP in dogs, cats, sheep, cattle, and horses measured by two tonometers TonoVet® and TonoVet Plus®. To the author’s knowledge, while obtaining data for this research, there has been similar research done on dogs and cats but no data on measurement differences in sheep and cattle.

Kiland *et al*. [5] compared IOP values in normal and glaucomatous cats, showing a strong correlation between TonoVet® and TonoVet Plus®. TonoVet® showed slightly higher IOP values than TonoVet Plus® [5]. Our results showed no significant differences between IOP values measured using TonoVet® and TonoVet Plus®. In a similar study conducted in dogs wherein IOP values in normal dogs and dogs with ocular diseases, including glaucoma and uveitis, were compared, higher values were measured using TonoVet Plus® than TonoVet® [4]. Similar to the results of our study and other previous studies, Guresh *et al*. [10] consistently measured significantly higher readings using TonoVet Plus®; however, these measurements did not exceed the expected IOP range in normal dogs [10]. The mean ± SD (range) IOP values obtained in the study were 15.0 ± 3.2 mmHg for TonoVet® and 19.2 ± 3.1 mmHg for TonoVet Plus®, which are similar to our results – 15.25 ± 2.77 with TonoVet® and 19.65 ± 3.46 with TonoVet Plus®. Although the values obtained were within the normal range, the values obtained using TonoVet Plus® were close to the upper limit set using the TonoVet tonometer and similar to those for TonoVet Plus, which can be confusing some clinical cases [4, 11].

In horses, the reported normal IOP values measured using TonoVet® and TonoVet Plus® were 14.4–27.2 mmHg for TonoVet® and 16.0–26.1 mmHg for TonoVet Plus®, with average values of 21.0 ± 3.14 mmHg for Tonovet® and 21.6 ± 2.45 mmHg for Tonovet Plus® [12]. In a study conducted with sedated horses and in a field study with non-sedated horses, a difference was seen between sedated and non-sedated horses, showing higher results in non-sedated horses but no significant changes between tonometers [13]. In our research, TonoVet® recorded lower IOP values compared to TonoVet Plus® (–2.13 mean difference). Horses in our research were not sedated but the IOP values we obtained were more similar to those of sedated horses (IOP TonoVet® 22.15 mmHg, TonoVet Plus® 24.28 mmHg in our research in non-sedated horses; TonoVet® 25.7 ± 5.8 mmHg, TonoVet Plus® 24.8 ± 7.1 mmHg in sedated and TonoVet® 30.7 ± 5.6 mmHg (range 21.7–38.0 mmHg); TonoVet Plus® 29.6 ± 6.7 mmHg) [13]. This may be due to studying different breeds and other external factors [13]. Latham *et al*. [14] compared TonoVet® and TonoVet Plus® measurements in horses with ophthalmic diseases and under auriculopalpebral block; both tonometers showed strong agreement; however, TonoVet Plus® also showed slightly higher measurements compared with TonoVet®.

To the best of our knowledge, no similar studies have measured IOP values in sheep and cattle. Several studies have investigated the average values of IOP measurements in different breed ewes but with different tonometers [15–17]. IOP values measured with TonoVet® vary between 11.7 ± 3.3 and 13.1 ± 4.4 mmHg [15–18]. In the Latvian Darkhead breed used in our study, the reference interval was reported to be 16.92–19.18 mmHg in ewes using TonoVet®. By comparing two tonometers in sheep, we noticed that the average IOP measured using TonoVet® was 18.05 ± 3.54 mmHg and 22.49 ± 4.66 mmHg measured using TonoVet Plus®. Similar to that observed in dogs, significantly higher IOP was noted in sheep with a mean difference of 4.48. To the best of our knowledge, no previous study by Kovalcuka *et al*. [19] reported the difference between sheep tonometer measurements.

In cattle, the difference was not significant. IOP was 24.73 mmHg for TonoVet® and 23.60 mmHg for TonoVet Plus®, with a suggested reference range of 23.83–25.62 mmHg for TonoVet® and 22.65–24.55 mmHg for TonoVet Plus®

## Conclusion

This research shows species-specific results, showing significant differences between TonoVet® and TonoVet Plus® measurements in sheep, dogs, and horses. All values were within the normal IOP range. Nevertheless, for clinical and research purposes, the same tonometer should be used when monitoring IOP over time, evaluating responses to therapy, and/or when taking serial IOP measurements over time.

## Authors’ Contributions

LK: Planned and designed the study and drafted and revised the manuscript. LK and JV: Performed the IOP measurements. AM: Data analysis. All authors have read, reviewed, and approved the final manuscript.
